# Synergistic Neuroprotection of Artesunate and Tetramethylpyrazine in Ischemic Stroke, Mechanisms of Blood–Brain Barrier Preservation

**DOI:** 10.3390/ijms26167979

**Published:** 2025-08-18

**Authors:** Yan Liang, Shuoqiu Deng, Yu Li, Shuiqing Qu, Chengcheng Liu, Luqi Wang, Lina Chen, Tuo Liu, Yujie Li

**Affiliations:** 1 State Key Laboratory for Qualty Ensurance and Sustainable Use of Dao-di Herbs, Institute of Chinese Materia Medica, China Academy of Chinese Medical Sciences, Dongcheng District, Beijing 100700, China; 18635431938@163.com (Y.L.); dengshuoqiu98@163.com (S.D.); m18331590467@163.com (Y.L.); 15703410357@163.com (S.Q.); liuchengcheng0414@163.com (C.L.); lqwang@icmm.ac.cn (L.W.); lnchen@icmm.ac.cn (L.C.); 2 Artemisinin Research Center, China Academy of Chinese Medical Sciences, Beijing 100700, China

**Keywords:** ischemic stroke, BBB, artesunate, tetramethylpyrazine, TIMP1, MMP9

## Abstract

Artesunate (AS) and tetramethylpyrazine (TMP) have been proven to have therapeutic potential in ischemic stroke. Nevertheless, their synergistic treatment mechanisms and effectiveness remain unclear. A rat MCAO model was induced, and AS, combined with TMP, was administered intranasally to rats once a day for 3 days. The neurological severity scores, TTC staining, and H&E staining were implemented to analyze tissue injuries. Evans blue staining and immunohistochemistry of ZO-1, occludin, MMP-9, and TIMP-1 were implemented to evaluate the integrity of the blood–brain barrier (BBB). ELISA was used to detect the expression levels of inflammatory factors TNF-α and IL-10. TUNEL staining and the protein expression of Bax and Bcl-2 were used to evaluate the apoptosis of brain tissue cells. The core targets were predicted by network pharmacology and verified by the OGD/R cell model and siRNA in vitro. Results showed that nasal administration of AS and TMP significantly ameliorated ischemic-stroke-induced neurological dysfunction, BBB disruption, and cortical neuronal apoptosis. The protective mechanisms mainly included adjusting the expression and ratio of tight junction proteins TIMP-1 and MMP-9 in brain tissue, regulating the HIF-1α-VEGF pathway, and anti-inflammatory effects. This study provides experimental support for the further development and application of AS and TMP nasal combinations and provides the foundation for expanding the practical-application value of artemisinin and its derivatives.

## 1. Introduction

Stroke is primarily caused by the sudden rupture of cerebral blood vessels or a vascular obstruction impeding the blood flow to brain tissue [1,2]. Global Burden of Diseases, Injuries, and Risk Factors Study (GBD) data suggest that stroke remains the second main cause of death and the third leading cause of death and disability worldwide, and the incidence and mortality rates of stroke are constantly increasing [3]. The report from the World Stroke Organization (WSO), in collaboration with the Lancet Neurology Commission, also shows that if immediate measures are not taken without delay, the number of deaths caused by stroke is expected to increase by 50% (9.7 million annually) worldwide by 2025 [4]. Therefore, the global health crisis caused by stroke should be taken seriously and recognized.

Ischemic stroke constitutes 71% of all strokes and is primarily caused by a cerebral artery embolism resulting from atherosclerosis, a thromboembolism, or vascular injury [5]. Although IV-tissue plasminogen activator (IV-tPA) is the only treatment strategy for acute ischemic stroke until recently, many patients cannot obtain its ideal therapeutic effects because of the limitation of the treatment time window and potential bleeding risk of IV-tPA [6]. Moreover, this type of treatment often ignores the disruption to the blood–brain barrier (BBB) and nerve damage caused by ischemic stroke, leading to a poor prognosis in patients [7].

Ischemic stroke initiates complex pathophysiological alterations, notably BBB impairment [8], ion imbalance [9], oxidative stress [10], and mitochondrial damage [11], ultimately leading to the apoptosis and necrosis of neurons in the ischemic zone. More importantly, BBB damage is a considerable factor for cerebral edema, hemorrhagic transformation, and poor prognosis in patients with ischemic stroke [12,13]. Therefore, restoration of BBB integrity and function has a positive regulatory effect on the rehabilitation of patients with ischemic stroke [14].

Compelling evidence indicates that matrix metalloproteinases (MMPs) degrade extracellular matrix components, mediating brain tissue damage during ischemia/hypoxia. This MMP activity contributes to BBB disruption post-infarction, ultimately promoting brain edema and hemorrhage [15,16,17]. Pathological research on cerebral infarction has found that the serum levels of Matrix Metalloproteinase-9 (MMP-9) in patients are significantly higher than those of other types of MMPs [18]. Moreover, recent research indicates that MMP-9 is an effective target for treating cerebral infarction, and the drugs that inhibit MMP-9 expression could maintain the integrity of the BBB and reduce cerebral edema [19].

The tissue inhibitor of metalloprotease-1 (TIMP-1), an endogenous inhibitor of MMP-9, is secreted by macrophages and fibroblasts. It promotes endothelial cell proliferation, inhibits neuronal apoptosis, and stimulates angiogenesis [20,21]. Clinical studies have shown that the risk prediction of major prognosis could be significantly improved when TIMP-1 in serum is added to a conventional factor model [22]. Research shows that TIMP1 could restore interactions between astrocytic endfeet and the endothelium, as well as BBB integrity, indicating that TIMP-1 plays a significant role in protecting the BBB [23]. Many preclinical studies have also shown that the lack of TIMP-1 exacerbates BBB damage in animals with focal cerebral ischemia [24,25], and the ratio of MMP-9 to TIMP-1 is relevant to BBB injuries and water content in the brain than these two factors alone [26,27], while therapeutic interventions focused on TIMP-1 and its downstream signals may be valuable in the improvement of BBB function after brain injury and neurological diseases [28].

Current research indicates that certain bioactive monomeric components in traditional Chinese medicine protect against key pathological mechanisms in ischemic stroke and BBB injury. Notably, 2,3,5,6-tetramethylpyrazine (TMP), the primary active compound in Ligusticum chuanxiong Hort, improves blood circulation and disperses stasis, vasodilatory, anti-platelet agglutinating [29], antioxidant [30], and ameliorating microcirculation effects [31], and it is widely employed to treat cerebrovascular occlusion disorders, such as cerebral ischemia [32] and cerebral thrombosis [33]. Studies have verified that TMP could promote neurological recovery after cerebral injury by improving the integrity and function of the BBB [34,35,36]. Moreover, Artesunate (AS), a water-soluble derivative of artemisinin with good bioavailability, high efficiency, and low toxicity, has anti-inflammatory [37], BBB protective [38], antioxidant [39], immunomodulatory [40], antibacterial [41], antitumor [42] and other pharmacological activities, maintaining high concentrations in the nervous system. Our previous research has confirmed that the nasal administration of AS and TMP combination played an effective role in the protection of cerebral ischemic nerve injury caused by Plasmodium infection in experimental cerebral malaria (ECM) by reducing vascular obstruction, increasing cerebral blood flow, and promoting angiogenesis and axon growth [43]. Furthermore, red blood cells infected with malaria could block cerebral microvasculature and then induce ischemic brain injury in the development of cerebral malaria, which is similar to the pathogenesis of ischemic stroke [44], indicating that the combination of AS and TMP by the pernasal method may have certain therapeutic intervention effects on ischemic stroke by therapy of promoting blood circulation.

To explore the combined neuroprotective mechanism of AS and TMP, this study adopted a method combining the in vivo MCAO rat model with the in vitro oxygen–glucose deprivation (OGD) cell model.

## 2. Results

### 2.1. Combined AS and TMP Improved Survival Rate, Behavioral Scores, and Infarct Area in Rats with Experimental Stroke

**Figure 1:**
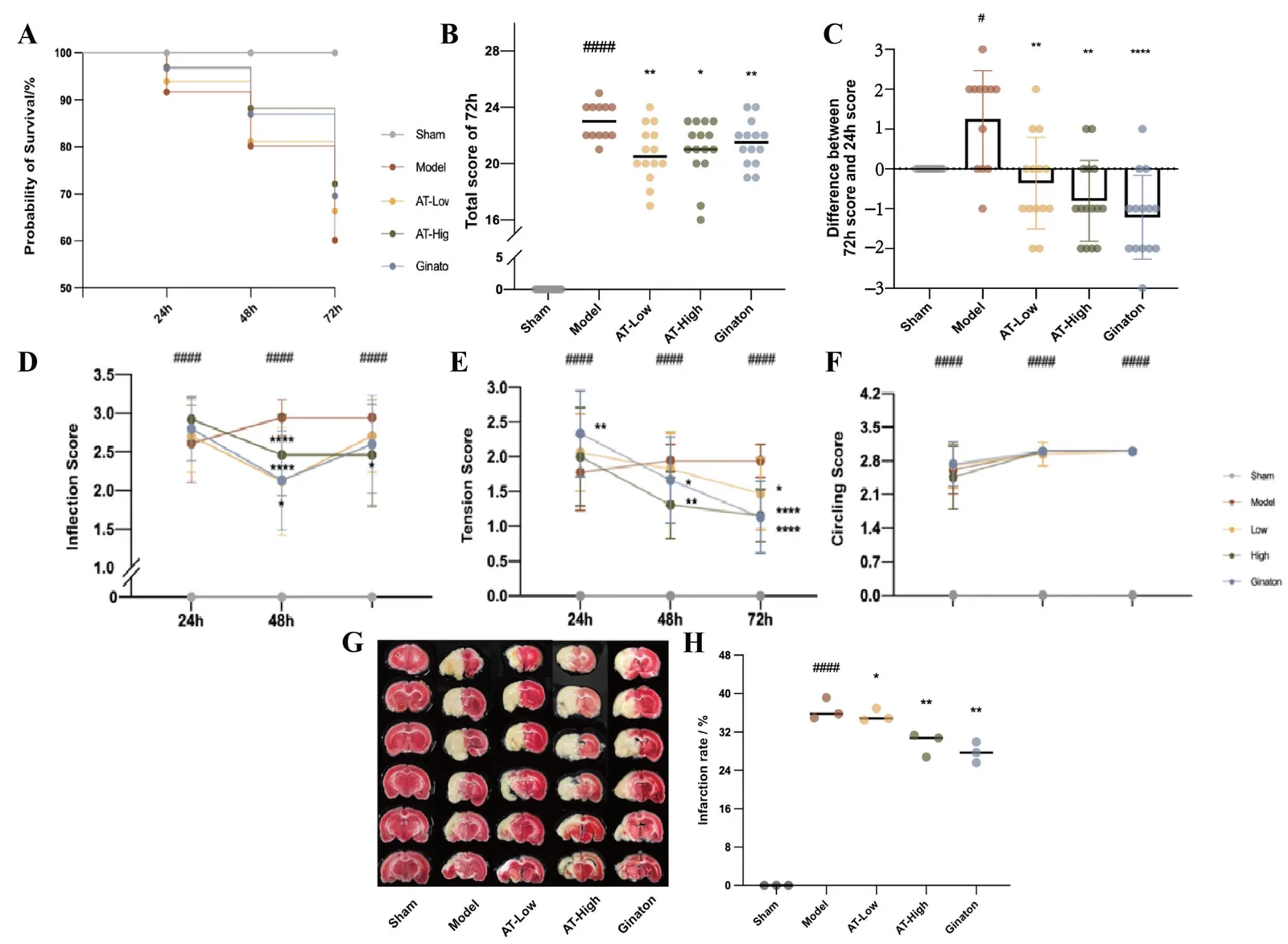
Combined AS and TMP improved the probability of survival, behavioral scores, and infarct area in rats with experimental stroke. (**A**) Probability survival. (**B**) Total score of 72 h. (**C**) Total score of 72 h. (**D**–**F**) Neurological behavior scores of inflections, tension, and movement track. (**G**,**H**) Infarct area. ^#^ *p* < 0.05 compared with sham group; ^####^ *p* < 0.0001 compared with sham group; * *p* < 0.05 compared with model group; ** *p* < 0.01 compared with model group; **** *p* < 0.0001 compared with model group.

To assess MCAO model validity and AS/TMP combination efficacy, we analyzed survival rates, behavioral scores, and infarct volumes. The mortality of rats in the model group was significantly higher than that of the sham group, while the mortality of rats in each treatment group at 24, 48, and 72 h was lower than that of the model group, and the AT-High group was the lowest (Figure 1A).

At the endpoint of the experiment (72 h after MCAO surgery), rats in the model group showed significant neurological injury. The total behavioral scores at 72 h after MCAO surgery and the difference between 72 h and 24 h of the rats in the model group were higher than those of the sham group (Figure 1B,C). Conversely, the total behavioral scores at 72 h after MCAO surgery and the difference between 72 h and 24 h of the rats in AT-Low, AT-High, and Ginaton groups were significantly decreased compared with the model group, suggesting that combined AS and TMP could alleviate nerve injury after MCAO surgery in rats. Compared with the sham group, the scores of the model group significantly increased at 24 h, 48 h, and 72 h. The neurological behavior scores of inflections and tension of each dose group were improved to some extent after administration (Figure 1D,E).

As shown in Figure 1G,H, the model group showed significantly larger infarct size compared with the sham group, while AT-Low, AT-High, and Ginaton could reduce infarct size obviously, indicating that ischemic injury was relieved after combined AS and TMP treatment.

### 2.2. Combined AS and TMP Improved Pathological Structure, Expression Levels of Inflammatory Cytokines, and Apoptosis of Brain Tissue in Rats with Experimental Stroke

**Figure 2:**
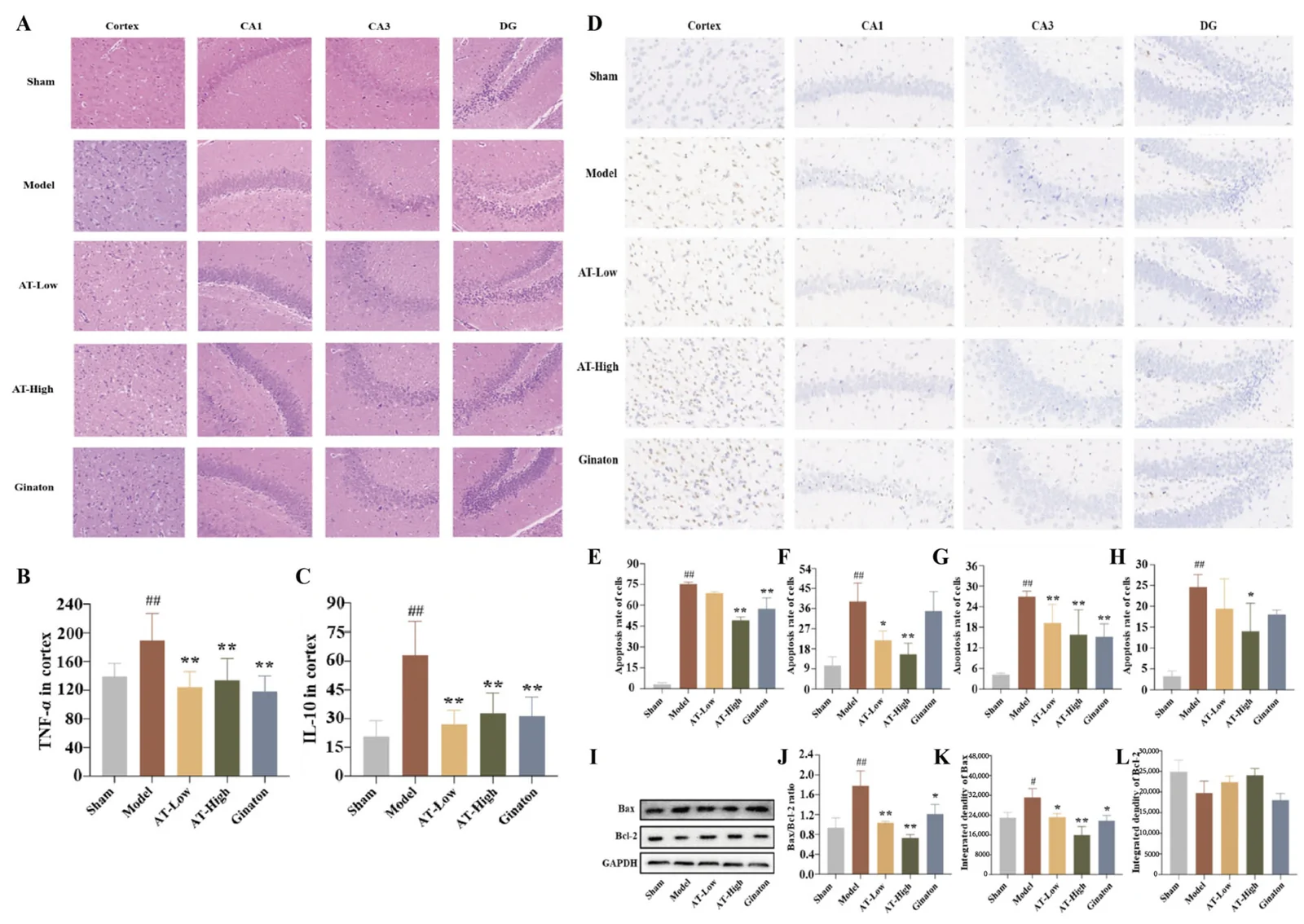
Combined AS and TMP improved MCAO-induced pathological structure injury, inflammation, and apoptosis of brain tissue in rats. (**A**) The H&E results were observed by a microscope (400×). (**B**) The level of TNF-α in the cortex. (**C**) The level of IL-10 in cortex. (**D**) TUNEL staining. (**E**) Apoptosis rate of cells in the cortex of each group. (**F**) Apoptosis rate of cells in the CA1 area of each group. (**G**) Apoptosis rate of cells in the CA3 area of each group. (**H**) Apoptosis rate of cells in the DG area of each group. (**I**) The Western blot bands. (**J**) Integrated density of Bax/Bcl-2. (**K**) Integrated density of Bax. (**L**) Integrated density of Bcl-2. GAPDH was used as a control. ^#^ *p* < 0.05 compared with sham group; ^##^ *p* <0.01 compared with sham group; * *p* < 0.05 compared with model group; ** *p* < 0.01 compared with model group.

HE staining results (400×, Figure 2A) indicated that rats in the sham group had a clear brain structure, the dense and orderly arrangement of nerve cells, and well-defined neuronal cell nuclei. However, rats in the model group had a sparse and irregular arrangement of nerve cells with a disordered structure and shrunken nucleus in brain tissue, and the pathological damage of the cortex was the most obvious. In contrast, the vacuolar changes in cells and nuclear shrinkage were reduced in the rats of each administration group.

The inflammatory levels were measured by detecting the levels of TNF-α and IL-10 in brain tissue. The levels of TNF-α and IL-10 were significantly higher in brain tissue than in the sham group, while there were lower levels of TNF-α and IL-10 in the AT-Low group and AT-High group compared with the model group, suggesting that MCAO surgery could induce inflammation of brain tissue, which could be improved by the treatment of combined AS and TMP (Figure 2B,C).

The apoptosis of neurons in infarcted side-brain tissue was quantitatively analyzed by TUNEL staining. There were few necrotic or apoptotic nerve cells in all the above areas of the sham group, while large amounts of apoptotic neurons (brown) were observed in each area of the infarcted hemisphere, and the cortex was the most obvious. Conversely, combined AS and TMP improved the apoptosis in each area. Moreover, the cell quantity in the cortex and hippocampus of the model group was reduced compared with the sham group, while the number of cells in the above areas of the AT-High group were higher than the model group, and cell quantity in CA1 and CA3 areas of AT-Low group were increased compared with model group (Figure 2D–H).

The Western blotting experiment was used to detect the expression levels of apoptosis-related proteins in the infarcted cortex. The protein expression of Bax in the model group was significantly increased compared to the sham group, while it was reduced after treatment in AT-Low and AT-High groups (Figure 2I–L). Although there were no significant differences among the sham group, model group, and administration groups, the ratio of Bax and Bcl-2 in the model group was higher than that in the sham group, and it was significantly decreased in AT-Low and AT-High groups. The results of TUNEL staining and Western blotting experiments suggest that AS and TMP, in combination, played an important role in improving MCAO-induced apoptosis of brain tissue in rats with experimental stroke.

### 2.3. Combined AS and TMP Improved Blood–Brain Barrier Injury in Rats with Experimental Stroke

**Figure 3:**
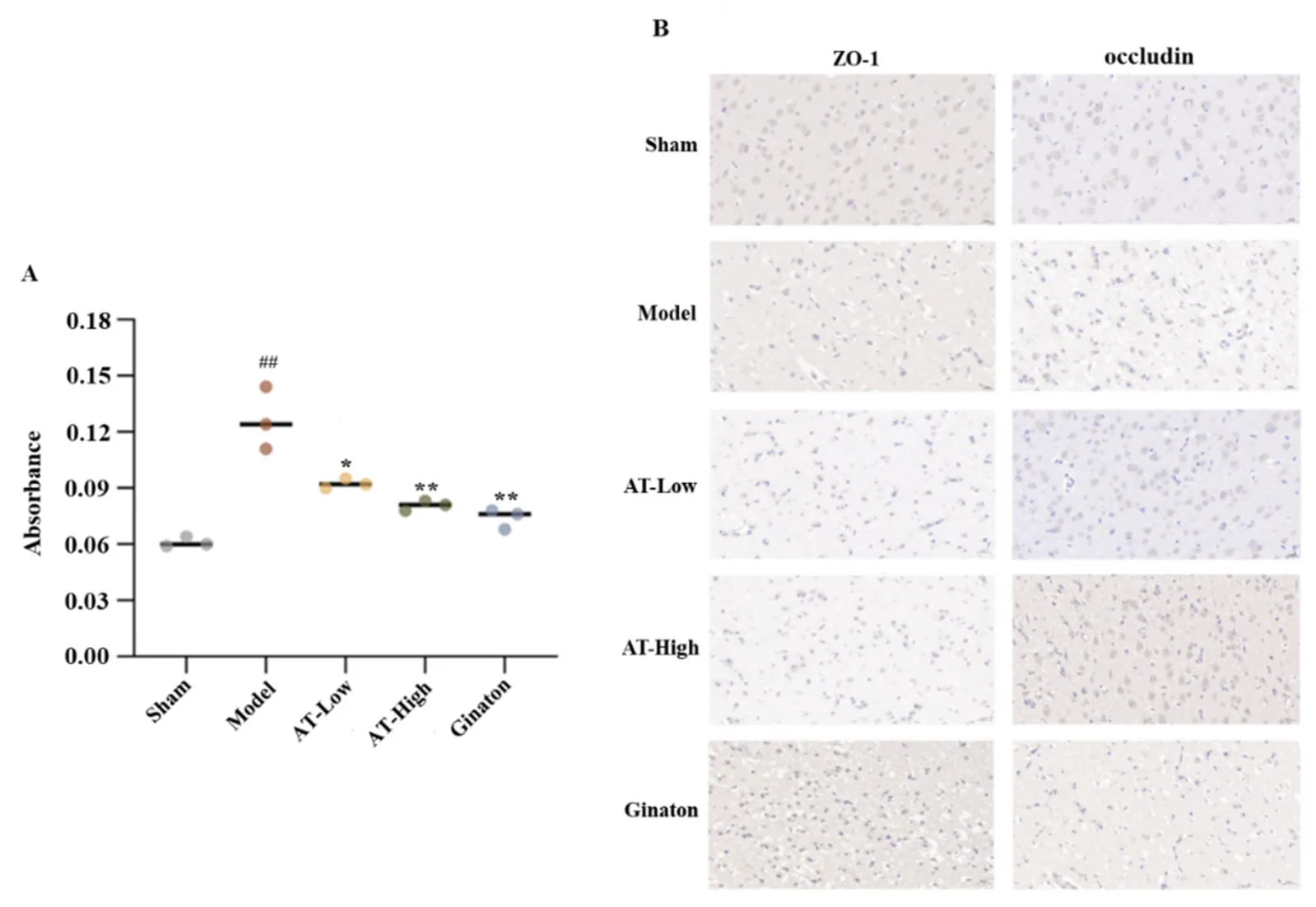
Combined AS and TMP improved BBB injury and destruction of the brain tissue in rats with experimental stroke. (**A**) Absorbance of Evans blue stain in each group. (**B**) The distribution and protein expression levels of tight junction protein ZO-1 and occludin were observed by immunohistochemistry. ^##^ *p* < 0.01 compared with sham group; * *p* < 0.05 compared with model group; ** *p* <0.01 compared with model group.

We evaluated the integrity of the BBB structure using the Evans blue extravasation method, and the absorbance value increased with the degree of BBB injury. The absorbance value of the model group was significantly higher than that of the sham group, suggesting that the BBB was damaged after the MCAO operation. Nevertheless, the absorbance value of each administration group was significantly lower than that of the model group, suggesting that the damage to the BBB was improved after the treatment of each drug (Figure 3A).

Immunohistochemistry was used to observe the distribution of ZO-1 and occludin and measure their protein expression levels. The brown positive areas of ZO-1 and occludin were predominantly distributed around the blue nucleus in the rats of the sham group, while the cell gap increased and the positive areas decreased in the model group. In AT-Low and AT-High groups, the cell gap decreased and the positive area increased, suggesting that combined AS and TMP could improve the MCAO-induced destruction of the tight junction structure in brain tissue (Figure 3B).

### 2.4. Network Pharmacology Results

#### 2.4.1. Integrative Analysis of “Clinical Practice Biomarkers Highly Recognized” for Stroke

**Figure 4:**
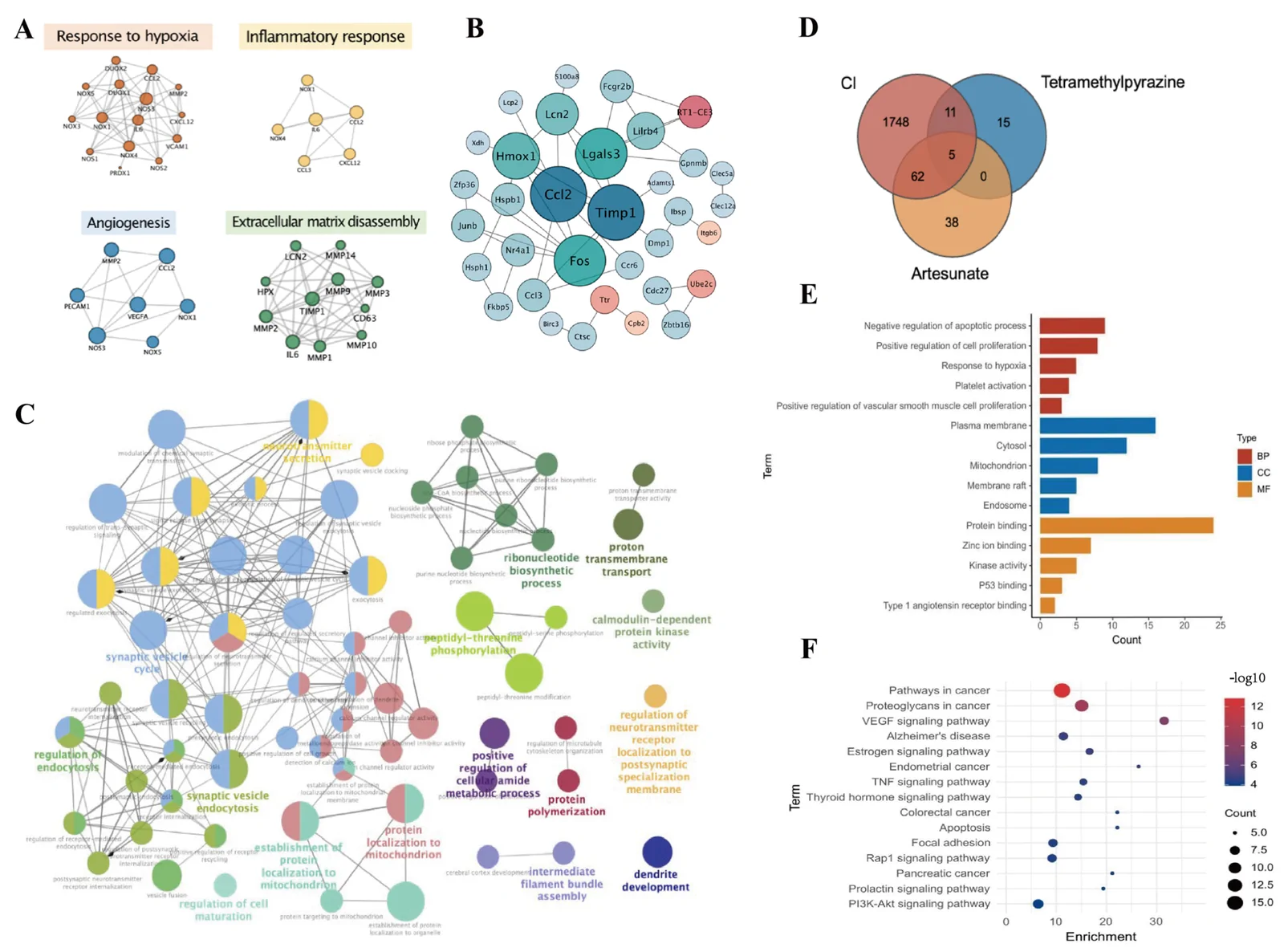
Prediction of the effects of AS and TMP intervention on progression of Cerebral Ischemia. (**A**) Analysis of a clinically highly recognized biomarker. (**B**) Integration analysis of transcriptome results. (**C**) Integrated analysis of proteome results. (**D**) Obtaining candidate targets. (**E**) GO analysis. (**F**) KEGG analysis.

A total of seven clinical reports on biomarkers of ischemic stroke were collected in the literature, including 6932 samples, and the biomarkers that were highly mentioned in them were sorted and summarized. Cluster analysis was performed with the help of the Gene Functional Classification module of the DAVID database. Biomarkers were mainly involved in four aspects of physiological processes, including responses to hypoxia, inflammatory responses, angiogenesis, and the decomposition of extracellular matrix. Moreover, protein interaction analysis was performed by the STRING website, and the results identified 37 highly identifiable biomarkers in key biological processes (Figure 4A).

#### 2.4.2. Establishment of “Multimaps Integrated Prediction” Network

In the first part of the network, we searched a total of 11 high-throughput transcriptomics pieces of literature on MCAO-induced ischemic stroke from the GEO database, and lgFC > 1 or <−1 was regarded as the unified standard to screen differential genes; then, 1132 upregulated genes and 2151 downregulated genes were obtained and brought into a subsequent network pharmacology analysis, and 35 common differential genes were obtained by intersecting the upregulated and downregulated genes using a Venn diagram. Protein–protein interaction analysis (PPI) was performed by the STRING database to obtain the interaction network of 35 differential genes, including 5 upregulated genes (pink) and 30 downregulated genes (blue). The results demonstrated that the TNF signaling pathway, ECM–receptor interaction, apoptosis pathways, and so on, were involved, and TIMP-1 was the node with the highest degree value (degree = 8), indicating that it interacted most closely with other genes among the aforementioned genes (Figure 4B).

In the second part of this network, we integrated and analyzed proteome sequencing data from the literature on ischemic stroke to obtain 193 significant differential proteins. As shown in Figure 4C, bioprocess (BP) analysis was performed by ClueGO v2.5.8 in Cytoscape. The main biological processes included the regulation of neurotransmitter secretion, dendrite development, the regulation of cell maturation, presynaptic endocytosis, and postsynaptic neurotransmitter receptor internalization.

#### 2.4.3. The Effect and Mechanism of Combined AS and TMP on Cerebral Ischemia Were Preliminarily Predicted

First of all, 31 targets of TMP and 105 targets of AS were acquired and analyzed based on network pharmacology. Second, the targets associated with “cerebral ischemia”, “ischemic cerebral injury”, and “ischemic stroke” were collected, and a total of 1826 disease targets were obtained after being merged and integrated. We took the overlapping targets between corresponding compound targets and disease targets, and a Venn diagram depicting intersecting targets is shown in Figure 4D. A total of 78 candidate targets, including caspase 3 (CASP3), angiotensin-converting enzyme (ACE), MMP9, caspase 1 (CASP1), VEGFA, and so on, were collected. PPI analysis (interaction score, 0.9) was used to combine the potential targets of AS, TMP, and the core targets. Cytoscape was used to analyze the PPI network, and 25 core targets were obtained. Finally, we successfully established a heterogeneous network of AS and TMP-induced cerebral ischemia.

Then, we collected the core targets and performed GO and KEGG analysis based on the DAVID 6.8 database. A total of 117 biological processes, 14 cell compounds, and 34 molecular functions were obtained (Figure 4E). The KEGG results indicated that 15 pathways were closely related to cerebral ischemia, including the VEGF signaling pathway, estrogen signaling pathway, TNF signaling pathway, apoptosis, and so on (Figure 4F).

### 2.5. Combined AS and TMP Improved MCAO-Induced Changes in TIMP-1 and MMP-9 Expression Levels

**Figure 5:**
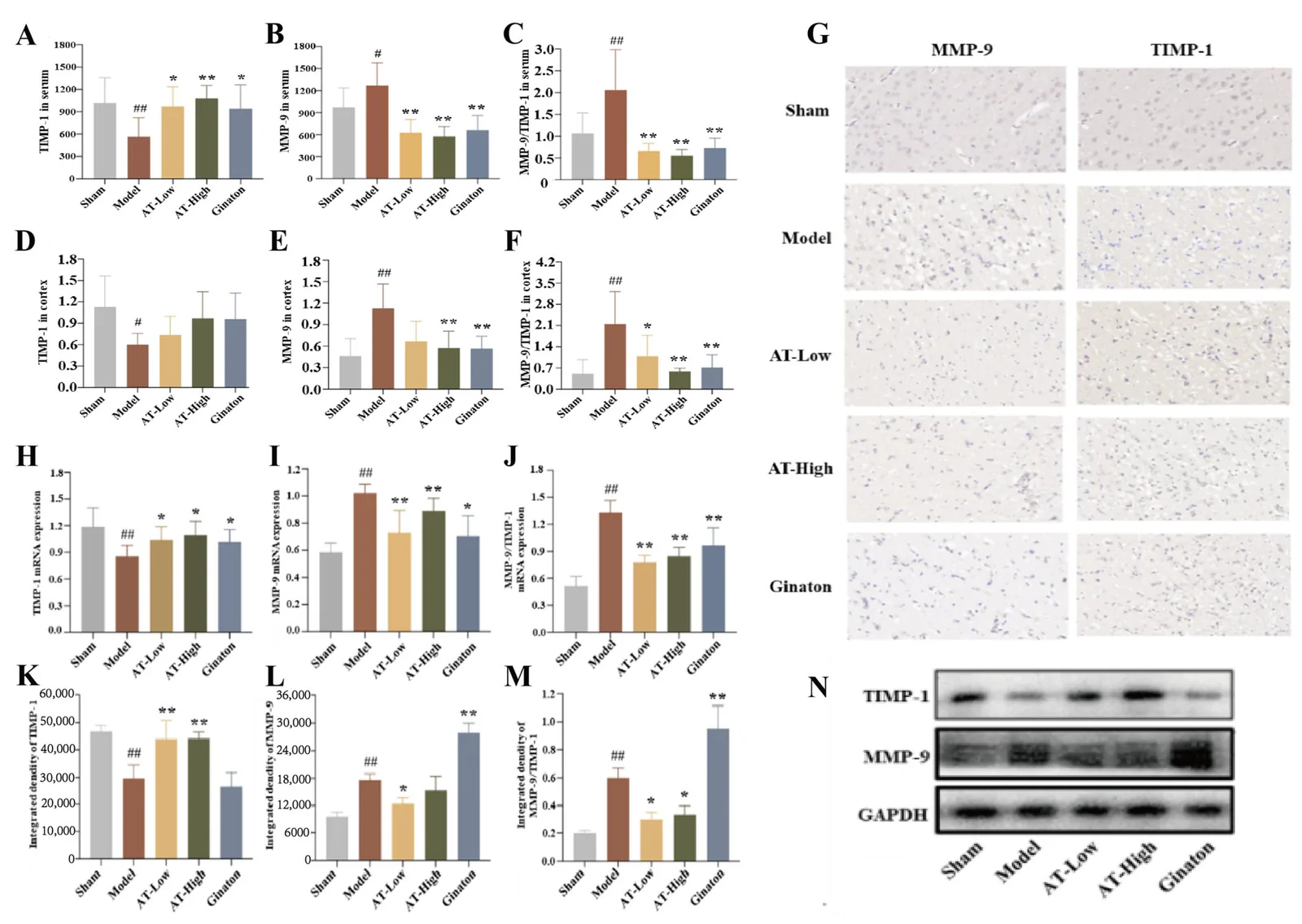
Combined AS and TMP improved MCAO-induced changes in TIMP-1 and MMP-9 expression levels. (**A**) The level of TIMP-1 in serum. (**B**) The level of MMP-9 in serum. (**C**) MMP-9/TIMP-1 ratio in serum. (**D**) The level of TIMP-1 in infarct side cortex. (**E**) The level of MMP-9 in infarct side cortex. (**F**) MMP-9/TIMP-1 ratio in infarcted side cortex. (**G**) Immunohistochemistry results of MMP-9 and TIMP-1 expression in brain tissue. (**H**) TIMP-1 mRNA expression. (**I**) MMP-9 mRNA expression. (**J**) MMP-9/TIMP-1 mRNA expression. (**K**) The protein expression of TIMP-1. (**L**) The protein expression of MMP-9. (**M**) The ratio of MMP-9 to TIMP-1 protein expression. (**N**) The Western blots bands. ^#^ *p* < 0.05 compared with sham group; ^##^ *p* < 0.01 compared with sham group; * *p* < 0.05 compared with model group; ** *p* < 0.01 compared with model group.

In vivo experiments and the levels of MMP-9 and TIMP-1 in serum and the infarcted cortex were determined through multiple strategies, including ELISA, immunohistochemistry, RT-PCR, and Western blotting, which were the key factors closely related to BBB injury in ischemic stroke. ELISA results showed that the level of TIMP-1 in serum was significantly decreased, while the level of MMP-9 and the proportion of MMP-9 and TIMP-1 were significantly increased in the model group. The levels of TIMP-1 were significantly increased, and the levels of MMP-9 and the proportion of MMP-9 and TIMP-1 were significantly decreased after AT and Ginaton treatment (Figure 5A–C). Moreover, ELISA results in the infarcted cortex (Figure 5D–F), RT-PCR results (Figure 5H–J), Western blotting results (Figure 5K–N), and immunohistochemistry results (Figure 5G) were also similar to the above results.

### 2.6. Combined AS and TMP Protected Cells Against Oxygen–Glucose Deprivation-Induced Cytotoxicity

**Figure 6:**
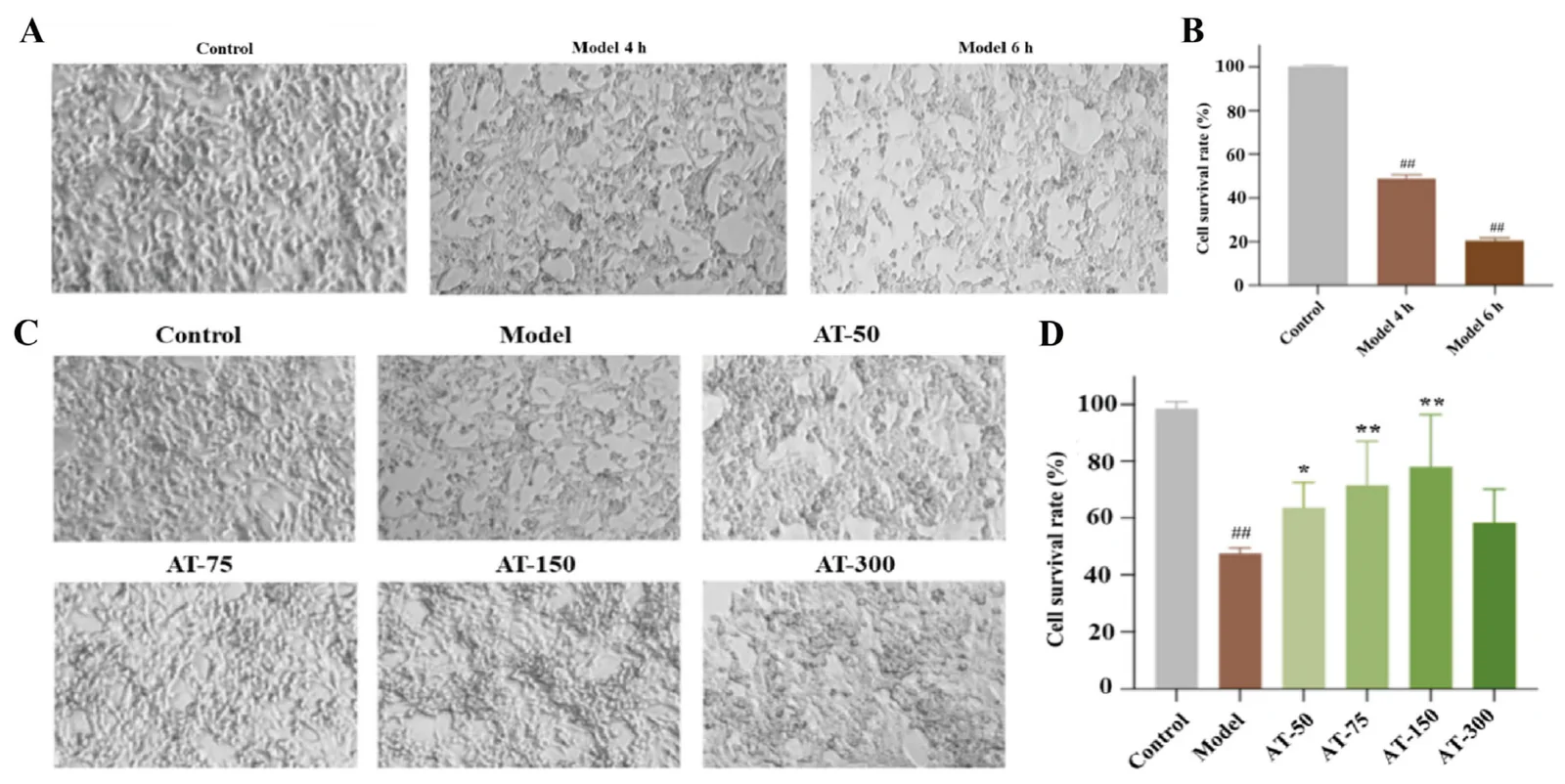
Combined AS and TMP had a protective effect on cell damage caused by OGD modeling. (**A**) Cell morphology. (**B**) CCK-8 results. (**C**) Cell morphology of each group. (**D**) CCK-8 results after drug administration. ^##^ *p* < 0.01 compared with control group; * *p* < 0.05 compared with model group; ** *p* < 0.01 compared with model group.

An OGD model was established to simulate ischemia/hypoxia stimulation at the cellular level, and it was used to evaluate the efficacy of the AS and TMP combination on cell damage. Results showed that cells in the control group had uniform distribution and full morphology, while the shape of the cells in the OGD group was abnormal, manifested as cell shrinkage. Moreover, the cell quantity in the model group significantly decreased, and the survival rate was reduced at 4 h and 6 h after OGD modeling (48.91% and 20.59%, respectively) (Figure 6A,B). On the contrary, 50, 75, and 150 μM of AT could increase the survival rate of cells effectively (Figure 6C,D), indicating that AS combined with TMP has an apparent protective effect on cell damage caused by OGD.

### 2.7. Combined AS and TMP Improved OGD-Induced Changes in TIMP-1 and MMP-9 Expression Levels in SK-N-BE Cells

**Figure 7:**
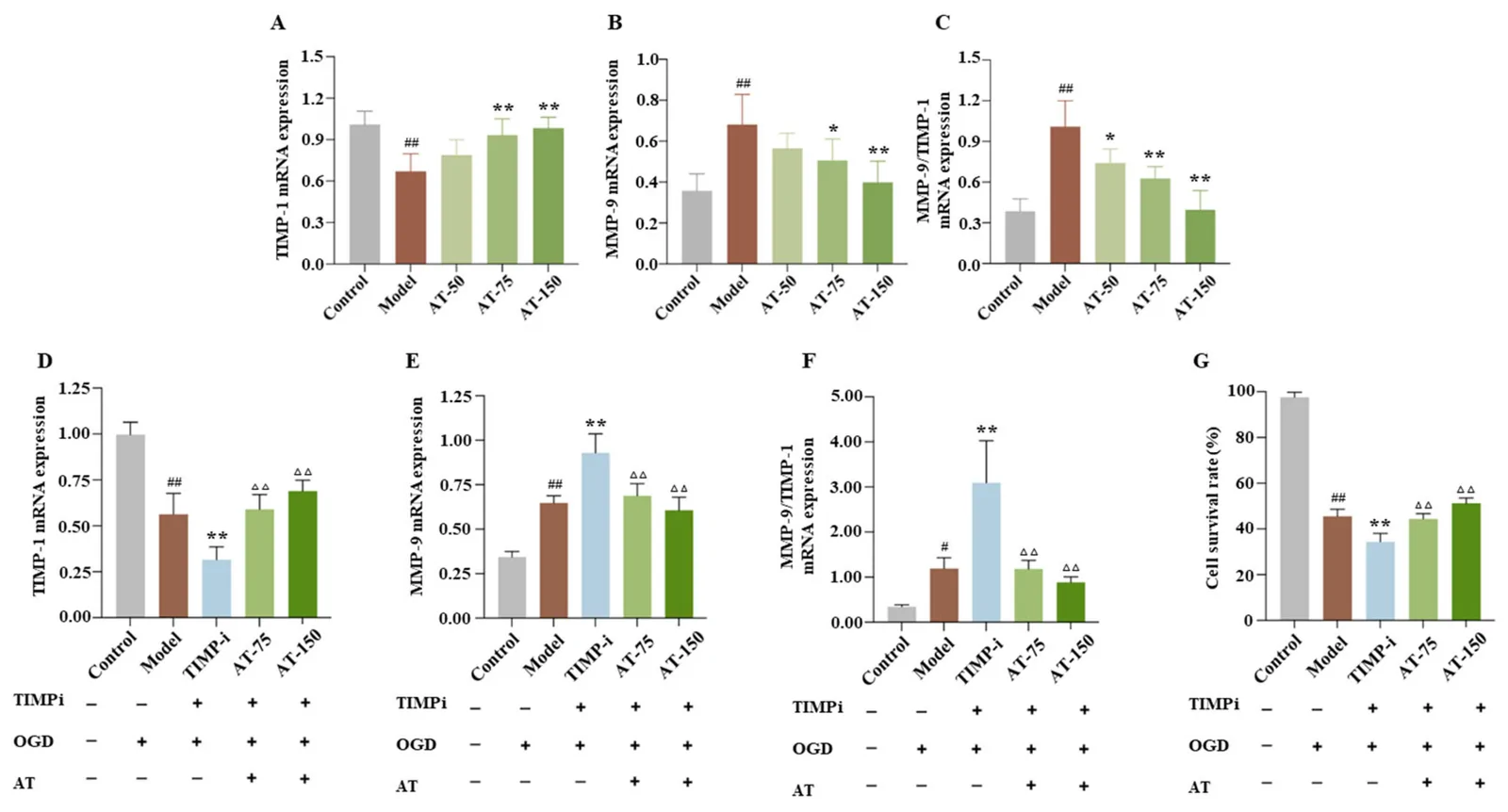
TIMP-1 played a crucial role in the mechanism of combined AS and TMP treatment for OGD injury in SK-N-BE cells. (**A**,**D**) TIMP-1 mRNA expression. (**B**,**E**) MMP-9 mRNA expression. (**C**,**F**) MMP-9/TIMP-1 mRNA expression. (**G**) Cell survival rate. ^#^ *p* < 0.05 compared with sham group; ^##^ *p* < 0.01 compared with sham group; * *p* < 0.05 compared with model group; ** *p* < 0.01 compared with model group; ^ΔΔ^ *p* < 0.01 compared with TIMP-i group.

During in vitro experiments, the mRNA expression of TIMP-1 in the OGD group was significantly reduced, and the mRNA expression of MMP-9 and the ratio of MMP-9 to TIMP-1 were significantly increased compared with the control group, while 75 and 150 μM were improved (Figure 7A–C).

In order to further investigate whether TIMP-1 played an essential role in the mechanism of combined AS and TMP treatment for ischemic stroke, siRNA technology was used to interfere with the TIMP-1 gene in OGD cell models. The TIMP-i group had a significant decrease in the TIMP-1 mRNA expression level and a significant increase in the MMP-9 mRNA expression level and MMP-9/TIMP-1 ratio, while these indicators showed varying degrees of callback after combined AT treatment (Figure 7D–F). Moreover, the cell survival rate of the TIMP-i group was lower than the model group, while that of the AT-50 group and AT-150 group significantly increased (Figure 7G), indicating TIMP-1 played a crucial role in the mechanism of combined AS and TMP treatment for OGD injury, and it is expected to become an important target for developing innovative drugs for treating cerebral ischemia in the future.

### 2.8. Combined AS and TMP Improved MCAO-Induced Changes in HIF-1α and VEGF Expression Levels

**Figure 8:**
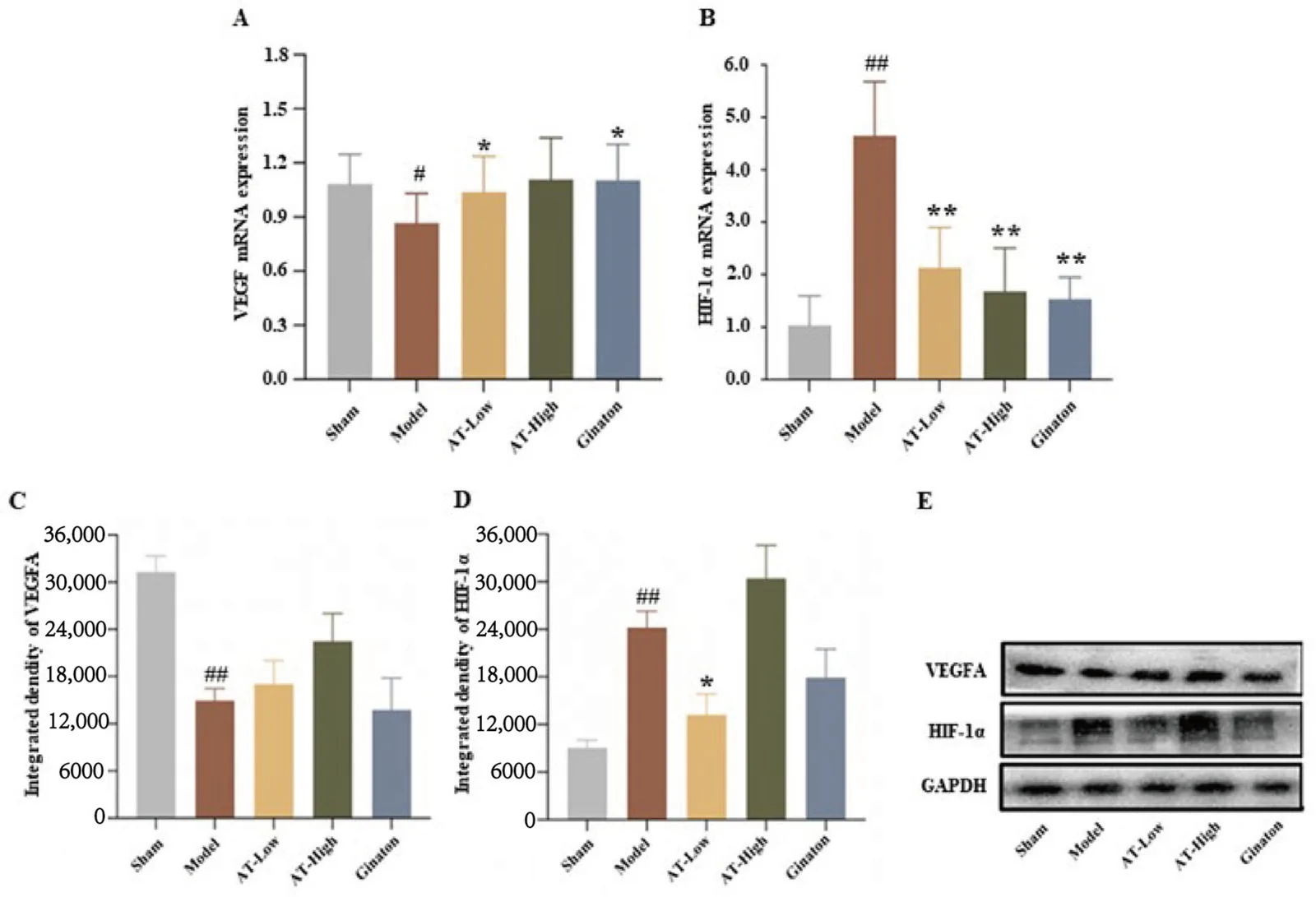
Combined AS and TMP improved MCAO-induced changes in HIF-1α and VEGF expression levels. (**A**) VEGF mRNA expression. (**B**) HIF-1α mRNA expression. (**C**) VEGF protein expression levels. (**D**) HIF-1α protein expression levels. (**E**) Western blot bands. ^#^ *p* < 0.05 compared with sham group; ^##^ *p* <0.01 compared with sham group; * *p* < 0.05 compared with model group; ** *p* < 0.01 compared with model group.

To further investigate the mechanism of combined AS and TMP improving cerebral ischemia, we detected the mRNA (Figure 8A,B) and protein expression of HIF-1α and VEGF in the infarcted cortex (Figure 8C–E) based on the network pharmacology results.

Compared with the sham group, the mRNA and protein of VEGF in the model group decreased significantly, while the mRNA expression of VEGF increased after the treatment of a low dose of AT (Figure 8A). However, AS and TMP administration could have increased the protein expression of VEGF, but there was no significant difference (Figure 8C). The mRNA and protein expression of HIF-1α in the model group significantly increased compared with the sham group, while the low dose of AT could have decreased the mRNA and protein expression of HIF-1α, and the high dose of AT could have decreased the mRNA expression of HIF-1α (Figure 8B,D).

## 3. Discussion

Over the past two decades, research on ischemic stroke (IS) has mainly concentrated on blood flow reconstruction [45], including thrombolytic therapy [46] and mechanical thrombectomy (MT) [47]. However, all of the above-mentioned therapies are limited by strict time windows (tPA ≤ 4.5 h, MT ≤ 6 h), and there are two major clinical challenges:, approximately 50% of the patients did not show a significant improvement in neurological function after vascular recanalization. Reperfusion injury may aggravate brain tissue damage [48,49]. The expansion of the time window and the long-term recovery of neurological function after thrombolysis have always been the core directions of treatment optimization. The latest clinical evidence indicates that prophylactic administration of the antibiotic minocycline can extend the treatment time window to 24 h after stroke and simultaneously reduce the risk of pneumonia complications [50]. The time window for alteplase (tPA) in ischemic stroke was also extended to 24 h, and the proportion of patients achieving functional independence through the modified Rankin Scale score at 90 days was higher [51]. As a consequence, the reduction in reperfusion injury and exploration of scientific and effective drugs are key clinical issues that urgently need to be addressed in current research on the prevention and treatment of ischemic stroke. Although this study revealed the mechanism by which AS and TMP administered together could alleviate damage to the BBB, it did not assess the impact of the treatment time window. This is one of the shortcomings of this study. In the future, experiments need to be designed to verify the efficacy attenuation point, clarify the mechanisms, and overcome the time-related bottleneck.

In recent years, more and more scholars have been devoted to studying the therapeutic effects of natural drugs on cerebral ischemia, especially natural drugs derived from plants, which have regulatory effects on various pathological processes such as oxidative stress, inflammatory cascade reactions, and cell apoptosis [52,53]. Our previous research confirmed that nasal administration of AS and TMP could significantly improve the survival rate of experimental cerebral malaria mice, and they had a synergistic protective effect on ischemic brain injury, which can alleviate neurological damage symptoms by protecting the BBB structure [43]. Based on the theory of homotherapy for heteropathy in Traditional Chinese Medicine, we proposed, as the hypothesis of this study, that nasal administration of AS and TMP would also have a protective effect on ischemic stroke with BBB damage and neurological sequelae caused by blood vessel blockage. Our results confirmed this hypothesis and demonstrated that combined AS and TMP treatment could effectively improve ischemic stroke caused by MCAO surgery, mainly manifested by improving the overall state and survival rate of MCAO model rats, reducing the infarct rate, alleviating brain pathological damage and BBB integrity, and reducing cortical nerve cell apoptosis.

Many studies show that after cerebral ischemia induces neurological damage, oxidative stress and inflammatory responses could destroy the structure and function of the BBB, resulting in vasogenic edema, which is prone to disability in severe cases [54,55]. BBB integrity critically determines functional recovery in cerebral ischemia. Network pharmacology predicted BBB protection as a key mechanism for AS/TMP combination therapy, which we validated experimentally. Evans Blue extravasation assays and immunohistochemical analysis of ZO-1 and occludin confirmed that AS combined with TMP ameliorates MCAO-induced BBB disruption and normalizes junctional protein distribution.

TIMP-1 and MMP-9 are key factors closely related to BBB injury in ischemic stroke [55]. TIMP-1 encodes proteins that are natural inhibitors of MMPs, and it is a group of peptidases involved in extracellular matrix degradation and has anti-apoptotic functions [56,57]. Clinical studies have shown that TIMP-1 can be applied to treat BBB dysfunction in brain injury [58,59], and in experimental stroke models, the lack of TIMP-1 exacerbates the disruption of the BBB [25,55,60]. In addition, clinical studies have shown that increased synthesis and activity of MMP-9 may lead to BBB dysfunction and brain edema, which can serve as a criterion for BBB injury [61,62]. The abnormal expression of MMPs and TIMPs can lead to tight junctions and disruption of the basal layer [21,63], and the ratio of TIMP-1 and MMP-9 has a strong correlation with BBB injury [64,65] and brain water content.

Our results showed that combined AS and TMP could significantly increase the levels of TIMP-1 and decrease the levels of MMP-9 and the proportion of MMP-9 and TIMP-1, which further indicated that combined AS and TMP had a certain protective effect on MCAO-induced BBB damage.

We also focused on the role of VEGFA in the combined AS and TMP treatment of experimental cerebral ischemia, as this target was identified in the results of network pharmacology. VEGFA, the sole endothelial-specific growth factor, potently induces angiogenesis and increases blood–brain barrier (BBB) permeability in vivo [66,67]. Studies have reported that the expression of VEGFA and its receptors was significantly upregulated in ischemic cerebrovascular disease, which leads to BBB dysfunction by inducing tight junction protein decomposition, occludin transportation, and phosphorylation of occludin protein Ser490, thereby increasing the permeability of the BBB after cerebral ischemia and aggravating cerebral edema [68]. We detected the levels of VEGFA and its upstream transcription factor HIF-1α in the cortex, and the results showed that combined AS and TMP could significantly improve the reduction in VEGFA expression levels and the rise of HIF-1α, suggesting HIF-1α and VEGF may be involved in the mechanism of combined AS and TMP in improving cerebral ischemia.

Inflammation is also an important factor in BBB damage. Increased permeability of the BBB could lead to harmful molecules, such as inflammatory mediators, entering the brain, thereby causing an inflammatory response and increasing the destruction of the BBB [16]. Our results also indicated that combined AS and TMP could significantly improve brain inflammation caused by MCAO; the levels of TNF-α and IL-10 in serum and the brain were reduced after the treatment of combined AS and TMP, and TNF-α and IL-10 were considered the pro-inflammatory cytokines involved in inflammation and immune responses [69]. This result also suggests that multiple mechanisms may be involved in combined AS and TMP, improving the experimental cerebral ischemia process caused by MCAO.

Comprehensively, our results illustrate that the nasal administration of AS and TMP exhibits a synergistic protective effect on experimental cerebral ischemia, and the observed beneficial effects may be attributed to a proportional adjustment between TIMP-1 and MMP-9, the regulation of the HIF-1α-VEGFA pathway, and anti-inflammatory effects to attenuate BBB injury.

## 4. Materials and Methods

### 4.1. Materials

Animals: The experiments were performed on adult male Sprague Dawley rats (SPF grade, 8 weeks of age, 280–300 g). All animals were obtained from the China National Institutes for Food and Drug Control (Beijing, China; Certificate No. SCXK2017-0005). Rats were acclimated for one week to standardized environmental conditions (25 ± 1 °C; 55 ± 5% relative humidity; 12 h light–dark cycle) prior to experiments. An adequate standard of food and water was provided. All animal procedures adhered to China’s Ministry of Health Animal Management Regulations (Decree No. 55, 2001) and were approved by the Laboratory Animal Center, Institute of Basic Theory for Chinese Medicine, China Academy of Chinese Medicine Science, Beijing, China (project identification code, IBTCMCACMS21-2202-03).

Cells: The SK-N-BE cells were cultured in a DMEM medium (WISENT, Nanjing, China) with a 10% FBS solution (WISENT, Nanjing, China). Moreover, cells were placed in a culture incubator containing 5% CO_2_ and 95% O_2_ at 37 °C.

Drugs: AS for injections was purchased from Guilin Pharmaceutical Co., Ltd. (Guilin, China, batch number ZA1190208), prepared before use, and used within half an hour. TMP for injections was purchased from Shijiazhuang No.4 Pharmaceutical Co., Ltd. (Shijiazhuang, China, batch number 18050701). Ginaton for injections (positive drug) was purchased from Chi Sheng Pharma & Biotech Co., Ltd. (Taipei, Taiwan, batch number M8218).

### 4.2. Preparation of Rat Ischemic Stroke and Drug Administration

An experimental cerebral ischemic stroke model was established in rats by MCAO [70]. The right external carotid artery (ECA) and internal carotid artery (ICA) of each rat were carefully exposed after intraperitoneal anaesthetization with 30mg/kg pentobarbital sodium. A monofilament nylon suture (diameter of 0.26 mm) with a spherical tip (diameter of 0.36  ±  0.02 mm) was inserted into the ECA to block the flow of blood to the middle cerebral artery. Meanwhile, rats in the sham group were treated with the same surgical procedure, except for the insertion of the monofilament. Rats were divided into five groups: sham operation (sham, *n = 12*), MCAO operation with saline treatment (model, *n = 20*), MCAO operation with Ginaton treatment (Ginaton, 10 mg/kg, *n = 20*), a low dose of AS and TMP treatment (AT-Low, clinical equivalent dose, 3.05 mg/kg AS and 7.2 mg/kg TMP, *n = 20*), and a high dose of AS and TMP (AT-High, twice the clinical equivalent dose, 6.09 mg/kg AS and 14.4 mg/kg TMP, *n = 20*). After surgery, rats were given corresponding drugs by nasal instillation once a day for three consecutive days (50 μL/animal), while rats in the sham and model groups were treated with the same volume of saline.

### 4.3. Survival Rate and Behavioral Assessment

At 24, 48, and 72 h after MCAO surgery, the survival rate was recorded in each group. Additionally, behavioral assessments were performed at the above points in time according to neurological severity scores (NSS) [71,72]. NSS was divided into three small experiments: the flexion experiment, the tension experiment, and the movement track experiment. The NSS scoring criteria are shown in Table 1. Rats with a score of 0 at 24 h were excluded (to avoid false positive results, animals with no neurological deficits (score = 0) must be excluded as they indicate unsuccessful occlusion) [73]. The rats that ultimately survived and whose MCAO model was successfully established were randomly divided into the model (*n = 7*), AT-Low (*n = 14*), AT-High (*n = 15*) (The combination of AS and TMP is referred to as AT), and Ginaton groups (*n = 14*, Positive drug).

At 72 h after MCAO operation, after anesthesia, blood collection, and perfusion, brain tissues (*n = 3*) were taken and fixed with 4% paraformaldehyde for 24 h. Paraffin embedding was performed. Continuous coronal sections (with a thickness of 5 μm) were made in the area 3 mm before and after the fontanelle to obtain paraffin sections. Subsequently, HE staining, TUNEL staining, and immunohistochemical staining were carried out.

### 4.4. Evaluation of Infarct Size in Brain Tissue via TTC Staining

At 72 h after the MCAO operation, brain tissue (*n = 3*) was separated and placed in a brain slicer. The first blade was placed coronally at the intersection of the optic nerve; two slices were cut upwards, and four slices were cut downwards at a distance of 2 mm between every two pieces. All slices were incubated in 2% triphenyl-2,3,5-tetrazolyl chloride (TTC, Merck-Sigma, Darmstadt, Germany) for 30 min and then soaked in 4% paraformaldehyde for 24 h. Image J (ImageJ bundled with 64-bit Java 8) software was used to analyze the infarct area.

### 4.5. Histopathological Observation

At 72 h after MCAO operation, brain tissue (*n = 3*) from all experimental groups underwent 24 h fixation in 4% paraformaldehyde. After paraffin embedding and sectioning, sections were baked and subjected to hematoxylin and eosin (H&E) staining. Subsequent dehydration, clearing, and mounting were performed. Finally, the pathological conditions of the sectioned tissues were observed under a microscope (Nikon, Tokyo, Japan) and photographed for the record.

### 4.6. Evaluation of BBB Integrity with Evans Blue Stain

The Evans blue (Sinopharm Group Chemical Reagent Co., Ltd., Beijing, China) stain extravasation method was used to evaluate the integrity of the BBB. At 72 h after MCAO surgery, rats (*n = 3*) were injected with 2% Evans blue stain via the tail vein at a dose of 4 mL/kg. At 2 h after circulation, precooled physiological saline was perfused into the left ventricle. The ischemic hemisphere was separated, immersed in 3 mL formamide, and then incubated at 37 °C for 24 h. The absorbance at the wavelength of 620 nm was measured by an enzyme labeling instrument (Molecular Devices, New York, NY, USA).

### 4.7. Immunohistochemical Staining

At 72 h post-MCAO, brain tissues were harvested from anesthetized rats (*n = 3* per group) and sectioned at 3.5 μm thickness using a microtome (Leica, Wetzlar, Germany). Following dewaxing and rehydration, sections underwent antigen retrieval via microwave irradiation at medium power for 10 min in a retrieval solution, were cooled naturally, and then rinsed in PBS with agitation. Endogenous peroxidase activity was quenched before 30 min of blocking. Sections were then incubated overnight at 4 °C with primary antibodies against zonula occludens-1 (ZO-1) (1:500), occludin (1:500), MMP-9 (1:500), and TIMP-1 (1:300). After secondary antibody incubation (50 min, RT), a DAB working solution was applied within hydrophobic barriers until an optimal brown–yellow positivity emerged under microscopic monitoring. The reaction was terminated by water rinsing, followed by hematoxylin counterstaining (3 min) and differentiation (10 s). Finally, sections were dehydrated and mounted for analysis using Image J software.

### 4.8. Evaluation of Neuronal Apoptosis via TUNEL Staining

At 72 h after MCAO operation, brain tissue (*n = 3*) was separated. Rat brain tissues were fixed with 4% paraformaldehyde and then routinely embedded in paraffin and sectioned into 4 μm sections. Apoptotic neurons were detected using the TdT-mediated dUTP Nick End Labeling (TUNEL) staining kit. TUNEL-positive nuclei with chromatin condensation and fragmentation were considered to represent apoptosis. The Image J software was used to count the number of TUNEL-positive cells. At least three perspectives from the infarcted brain tissue area of each rat were selected for analysis, and the average value of these perspectives was taken as the final value of the rat.

### 4.9. ELISA Analysis

At 72 h after MCAO operation, the blood samples of rats without injection of Evans blue were collected by the aorta abdominalis method and centrifuged at 4 °C, 3000 rpm to obtain serum. The infarcted cortex was homogenized at 4 °C, centrifuged, and the supernatant was taken for follow-up experiments. Enzyme-Linked Immunosorbent Assay (ELISA) kits were used to measure the TNF-α, IL-10, TIMP-1, and MMP-9 levels in the serum (*n = 10*) and cortex (*n = 6*).

### 4.10. Network Pharmacology Analysis

Disease targets collection: through the NCBI database (https://www.ncbi.nlm.nih.gov/, accessed on 20 February 2023), screening of ischemic cerebral apoplexy clinical biomarkers; based on the GEO database, the research of the screening type was “Expression profiling by high throughput sequencing”; research on proteomic sequencing was collected based on databases such as NCBI, the Web of Science and CNKI. The above data were screened for differential genes based on the criteria of log2FC > 1 or <−1. With the help of the DAVID database, the ENSUMLE numbers were converted into OFFICIAL GENE Symbols, and the species were uniformly corrected to obtain all the differential proteins, which were simultaneously used for subsequent network pharmacological analysis. We counted the occurrence times of each protein in the sequencing results and collected the key differential proteins. Through GeneCards (https://www.genecards.org/, accessed on 20 February 2023) and OMIM (https://www.omim.org/, accessed on 20 February 2023), collection targets were associated with ischemic stroke.

Drug target collection: The related drug targets of AS and TMP were screened and predicted by using TCMSP, SwissTargetPrediction, and PharmMapper.

Establish PPI network: We used the Uniprot (https://www.uniprot.org/, accessed on 21 February 2023) database to correct the Gene name and Gene ID. The intersection of ischemic stroke with AS and TMP was selected as a candidate target for drawing a Venn diagram. We input the intersection target into STRING (https://string-db.org/, accessed on 21 February 2023) to obtain the protein interaction network and the hidden break nodes in the network, and establish the PPI network by using Cytoscape 3.8.2. We calculated the median of three topological indicators: “degree”, “closeness of nodes”, and “betweenness of nodes”. The core target of all three values was to be greater than the median.

GO and KEGG functional enrichment analysis: DAVID 6.8 was used to conduct enrichment analysis of core target GO and KEGG pathways for candidate targets. Results with *p* < 0.05 were selected and ranked by Degree.

Network construction: To analyze the correlations among core targets, Cytoscape 3.8.2 was used to create the corresponding network diagrams of components, core targets, and pathways.

### 4.11. Establishment of the OGD Model and Treatment

An OGD model was established in SK-N-BE cells to investigate AS combined with TMP neuroprotective mechanisms. Model group cells underwent 4 h of hypoxia (1% O_2_ and 5% CO_2_) in a glucose/serum-deprived medium.

Cells in a natural growth state were treated with 10–1000 μM AS and 10–1000 μM for 12 h, and the CCK-8 method was used to measure the survival rate of cells to determine whether the drugs themselves caused damage to cells in the experiment, and to explore a reasonable range of drug concentration. Afterwards, the AS and TMP of 50, 75, 150, and 300 μM were chosen to treat OGD cells in the model group based on the results of CCK-8, and cells were divided into six groups: control, model, AT-50, AT-75, AT-150, and AT-300 groups. Except for the control group, all other groups used an OGD modeling process for 4 h, and drug-treated groups were given a corresponding dosage of drugs. The effects of AS and TMP on cell damage caused by OGD were evaluated by CCK-8, and all samples were collected at the experimental endpoint for subsequent experiments.

### 4.12. Real-Time Reverse Transcription Polymerase Chain Reaction

Total RNA was extracted from infarcted brain tissues and cellular samples using an animal tissue total RNA extraction kit (Gene–protein Link, Beijing, China), according to the manufacturer’s protocol. We used the reverse transcription pre-mix kit RT-Mix (First-generation reverse transcription master mix RT-Mix of two-step method, G03R01, Gene–protein Link, China) to perform reverse transcription and synthesize cDNA. Primer sequences were designed as shown in Table 2. The PCR program was as follows: 95 °C for 1 min, followed by 30 cycles of 94 °C for 30 s, 60 °C for 30 s, and 72 °C for 30 min, and a final elongation of 10 min at 72 °C. The relative quantitative (RQ) method was adopted to calculate the relative content of the target gene mRNA, using GAPDH as a reference gene. cDNA was extracted from the brains of the sham group rats as a control reference. The expression level of mRNA was calculated using the formula RQ = 2^−ΔΔCT^, where ΔΔC_T_ = (C_T_ target gene − C_T_ β-actin)_embolism group_ − (C_T_ target gene − C_T_ β-actin)_sham group_, (*n = 6*).

### 4.13. Western Blotting

We added 200 μL of the mixture of the tissue lysis buffer and protease inhibitor and used a tissue homogenizer to break up the infarcted side-brain tissue (*n = 6*). Following centrifugation to collect supernatants, protein concentrations were determined. Extracted proteins were then denatured prior to Western blotting. After electrophoresis, proteins from all groups were transferred to membranes. The membranes were blocked with 5% skimmed milk and incubated with primary antibodies against MMP9 (1:2000, Proteintech, New York, NY, USA), TIMP1 (1:3000, Proteintech, USA), VEGFA (1:1000, Proteintech, USA), and HIF-1α (1:10000, Proteintech, USA) overnight at 4 °C. The PVDF membranes were incubated with a secondary antibody (HRP-labeled goat anti-rabbit IgG, 1:5000, Gene–protein Link, China). The membranes were then washed with a TBST buffer, and the PVDF membrane was immersed in the ECL detection reagents (Merck Millipore, New York, NY, USA) and quantified on a FluorChem R Gel imaging and analysis system (Protein Simple, Inc., New York, NY, USA).

### 4.14. Small Interfering RNA Transfection

Cells in a 96-well plate were divided into a control group, model group, TIMP-1 interference group (TIMP-i group), low dose of AS and TMP group (75 μM, AT-75), and high dose of AS and TMP group (150 μM, AT-150). After transfection with TIMP-1 small interfering RNA (siRNA) for 6 h, the supernatant was replaced with a culture medium containing drugs, and the cells were continued to be cultured for 24 h. After OGD for 4 h, the cell survival rate was detected by CCK-8. Moreover, the same procedure was performed for 24-well plate cells, and the RNA from cells was collected after 4 h of OGD for subsequent experiments. The sequence of the RNA oligo is shown in Table 3.

### 4.15. Statistical Analysis

Data were expressed as the mean ± standard deviation (x ± s), and one-way analysis of variance (ANOVA) was used to analyze the data, presenting normal distribution with equality of variances using GraphPad Prism (version 9.0, San Diego, CA, USA). *p* < 0.05 was determined statistically significant.

## 5. Conclusions

The research results show that the combination of AS-TMP can improve the survival rate and NSS score of ischemic stroke model rats. It alleviated the pathological damage to brain tissue caused by ischemia. The combination of AS-TMP can improve the leakage of the BBB, increase the positive expression of ZO-1 and occludin, and reduce the intercellular space. The combination of AS-TMP reduces the number of apoptotic cells in the cortex and hippocampus, decreases the expression of Bax protein, and lowers the ratio of Bax/Bcl-2.

Based on network pharmacological analysis, the core targets of AS-TMP combination for anti-ischemic stroke are TIMP-1, VEGFA, MMP9, etc., which are mainly related to apoptosis, cell proliferation, and vascular smooth muscle cell proliferation, focusing on the VEGF signaling pathway.

The results of the mechanism study showed that the combination of AS-TMP could adjust the expression levels of TIMP-1 and MMP-9 in the animal serum and cortical brain tissue of rat models of ischemic stroke. Immunohistochemistry of brain tissue, RT-qPCR, and Western blotting in rat models of ischemic stroke all obtained the same results. In addition, the combination of AS-TMP increased the expression of VEGFA. In vitro experiments further verified the above results by interfering with Timp-1.

This study demonstrates that the combination of AS-TMP regulates the expression and ratio of MMP-9 and TIMP-1 and modulates the HIF-VEGF signaling pathway, thereby exerting a synergistic therapeutic effect of protecting the integrity of the blood–brain barrier and alleviating ischemic injury caused by stroke. In the future, it is necessary to conduct a co-culture of neural cells and vascular endothelial cells to better simulate the structure of the BBB in vivo, which can better observe the effects of the combination of AS-TMP on the BBB and further evaluate the mechanism of the combination.

## Figures and Tables

**Table 1:** Scoring table of severe nerve injury defect (NSS) in rats.

Score	Flexions	Tensions	Movement Track
0	Double forelimbs are symmetric to the ground	The muscle tone of both forelimbs is equal and powerful	Walk forward normally
1	Operation of the contralateral forelimb wrist flexion	The contralateral limb surgery tension shows a mild decline	Walking direction is slightly offset
2	Operation of the contralateral Shoulders internal rotation, and elbow flexion	The contralateral limb surgery tension shows a moderate decline	Keep turning on one side of the big circle
3	Both the shot buckling wrist and shoulder and internal rotation	The tension of the contralateral limb after the operation decreased severely	Keep turning small circles to one side

**Table 2:** Primer sequence.

RNA	Forward Primer	Reverse Primer
MMP-9	GAGACACGCTAGAGCAGATACC	TGGTCTCGATGATTTCTGGGG
TIMP-1	AGGGCCCCTTTCTTATTGCC	CACATTTTGCGCCCAGAGAA
VEGFA	AGAAAGCCCATGAAGTGGTGA	CTTCATCATTGCAGCAGCCC
HIF-1α	CATGATGGCTCCCTTTTTCA	ACATAGTAGGGGCACGGTCA
GAPDH	CCGCGAGTACAACCTTCTTG	CCGCGAGTACAACCTTCTTG

**Table 3:** Sequence of RNA oligo.

Oligo	Sense	Antisense
Negative control	UUCUCCGAACGUGUCACGUTT	ACGUGACACGUUCGGAGAATT
GAPDH Positive control	UGACCUCAACUACAUGGUUTT	AACCAUGUAGUUGAGGUCATT
TIMP oligo 1	GCAGCGAGGAGUUUCUCAUTT	AUGAGAAACUCCUCGCUGCTT
TIMP oligo 2	GCAAUUCCGACCUCGUCAUTT	AUGACGAGGUCGGAAUUGCTT
TIMP oligo 3	GGACUCUUGCACAUCACUATT	UAGUGAUGUGCAAGAGUCCTT

## Data Availability

The original contributions presented in this study are included in the article. Further inquiries can be directed to the corresponding authors.
